# Inaugural meeting of the malaria policy advisory committee to the WHO: conclusions and recommendations

**DOI:** 10.1186/1475-2875-11-137

**Published:** 2012-05-31

**Affiliations:** 1 Global Malaria Programme, World Health Organization, 20 Avenue Appia, CH-1211, Geneva 27, Switzerland

**Keywords:** Global, Malaria, Policy development, WHO, Drug resistance, Diagnostic tests, Mosquito control, Elimination, Surveillance, Chemoprevention

## Abstract

The Malaria Policy Advisory Committee to the World Health Organization met for the first time from 31 January to 2 February 2012 in Geneva, Switzerland. This article provides a summary of the discussions, conclusions and recommendations from that meeting, as part of the newly launched *Malaria Journal* thematic series “WHO Malaria Policy Advisory Committee: Reports and Recommendations”.

Summaries are provided, referencing the relevant background documents, for the meeting sessions on global malaria control, drug resistance and containment, rapid diagnostic test procurement criteria, larviciding, classification of countries for elimination, estimating malaria cases and deaths, and seasonal malaria chemoprevention. Policy statements, position statements, and guidelines that will arise from the MPAC meeting conclusions and recommendations will be formally issued and disseminated to World Health Organization member states by the World Health Organization Global Malaria Programme.

## Background

The Malaria Policy Advisory Committee [1] (MPAC) to the WHO met for the first time from 31 January to 2 February 2012 in Geneva, Switzerland [2]. This article provides a summary of the discussions, conclusions and recommendations from that meeting ^a^ as part of the newly launched *Malaria Journal* thematic series “WHO Malaria Policy Advisory Committee: Reports and Recommendations”, the prelude for which was published earlier [3].

The following sections of this article provide details and references for the background documents presented at the open sessions of the meeting on global malaria control, drug resistance and containment, rapid diagnostic test (RDT) procurement criteria, larviciding, classification of countries for elimination, estimating malaria cases and deaths, and seasonal malaria chemoprevention (SMC). The MPAC discussion and recommendations related to these topics, which took place partially in closed session, are also included. MPAC decisions are reached by consensus [3].

### Report from the WHO global malaria programme

The Director of the WHO Global Malaria Programme (WHO-GMP) opened the meeting with an overview of progress in global malaria control and elimination as reported in the *World Malaria Report 2011*[4,5], including a summary of the opportunities and major challenges ahead, such as sustaining political commitment, managing the projected decrease in programme funding, and increasing anti-malarial drug and insecticide resistance. Action by WHO-GMP, MPAC, and the global malaria community as a whole, will be critical in addressing these challenges.

The MPAC commended the increasing quality of each issue of the annual *World Malaria Report*, and strongly encouraged the continued commitment and participation of WHO member states in providing quality data to WHO-GMP to aid the accurate monitoring of progress against global malaria goals. They recommended strengthened linkage of the *World Malaria Report* to the African Leaders Malaria Alliance (ALMA) score card [6] to increase the feedback loop and engagement with WHO member states. The MPAC also highlighted the need for timely high quality data to guide malaria control that addresses local transmission factors, as opposed to a narrow focus on procurement of commodities, especially now that transmission is decreasing in many parts of the world [4].

### Drug resistance and containment

WHO-GMP presented an update on artemisinin resistance [7] in known foci in Cambodia and Thailand, and new suspected foci of resistance in Myanmar and Viet Nam, as well as a proposal for the establishment of a standing Technical Expert Group (TEG) to advise the MPAC specifically on drug resistance and containment and the implementation of the Global Plan for Artemisinin Resistance Containment (GPARC) [8]. The rationale for convening a standing TEG for this purpose is that the issue of anti-malarial drug resistance will need to be addressed with continued urgency, and that the topic is too large to fall within the remit of the existing TEG on chemotherapy or a new short-term Evidence Review Group (ERG).

The MPAC recommended that the scope of the TEG should cover broad aspects of drug resistance and containment, but that the draft terms of reference (ToR) appeared to focus heavily on confirmation of artemisinin resistance and monitoring. They advised that the scope of the TEG, and its membership, be broadened to reflect these needs, and to cover all regions, not just Asia. They also recommended that the proposed TEG on drug resistance and containment and the current standing TEG on chemotherapy should meet back-to-back, with an overlapping session when possible, as their areas of work are closely related. Each TEG’s recommendations will have an impact on those of the other TEG, which requires that their work be well co-ordinated.

The TEG will identify priorities for operational research. Initial priorities identified by MPAC members included the use of primaquine as a gametocytocide in the treatment for *Plasmodium falciparum* and the identification of molecular markers of artemisinin resistance.

The MPAC highlighted the issue of funding for and implementation of drug efficacy monitoring systems and suggested making better use of potential funding from the Global Fund for AIDS, Tuberculosis and Malaria (Global Fund) as a point of leverage, and that therapeutic efficacy monitoring could be required for grants that procured anti-malarial medicines. The Global Fund, a standing observer to the MPAC, raised the issue that making surveillance a key performance indicator had implications, namely it might result in withholding funding to countries where monitoring requirements are not adequately met, which would be at odds with the push to have anti-malarial medicines listed as life-saving commodities on a par with antiretroviral therapy. These are issues for the new TEG to consider.

The MPAC recommended that WHO-GMP should lead the global artemisinin resistance containment effort, and unanimously endorsed the creation of a TEG to provide on-going advice to the MPAC in how best to support the implementation of the GPARC.

The TEG ToR were reformulated in accordance with MPAC suggestions for improvement and presented back to MPAC before the end of the meeting; they were approved [9] pending minor corrections. WHO-GMP, in close consultation with the MPAC, will convene the TEG prior to the next MPAC meeting to conduct a critical review of global drug resistance monitoring.

### Rapid diagnostic test procurement criteria

WHO-GMP presented arguments for and against changing the WHO recommended RDT procurement criteria based on the WHO Malaria RDT Product Testing Programme ^b^ from a minimum threshold panel detection score (PDS) of 50%, set in 2009, to 75% for *P. falciparum* at low parasite densities (200 parasites/μL) in areas with high malaria transmission [10]. Of note, the PDS is a measure of product performance that is not the same as the sensitivity of the test in clinical settings ^c^.

The main arguments in favour of changing the threshold were that: (a) it will simplify procurement by aligning the detection thresholds used for both *P. falciparum* and *Plasmodium vivax* in all transmission settings; (b) a new threshold of 75% will be met by 21, as opposed to 24, *P. falciparum*-only RDTs, resulting in little change in terms of current product availability based on results of Rounds 1–3; (c) as malaria control improves, demand will increase for RDTs with PDS of at least 75% at low parasite densities for *P. falciparum* and *P. vivax*; and *(*d) there are immediate, theoretical benefits to individuals if a new threshold of 75% is implemented, particularly for vulnerable groups, such as asymptomatic pregnant women*.*

The main arguments against changing the threshold were that: (a) based on several recent studies in moderate to high transmission settings, there is no evidence that a PDS threshold of 50% for *P. falciparum* at low parasite densities (200 parasites/μL) is unsafe for patients; (b) the linear distribution of PDS, with small incremental differences and lack of confidence intervals, means that setting threshold levels could be perceived as arbitrary; and (c) as WHO-GMP does not systematically monitor which RDTs are currently in use in endemic countries and as Round 4 of the WHO Malaria RDT Product Testing is on-going, it is not known how changes in the threshold will impact existing practices and how many additional RDTs will be excluded from procurement based on the proposed change in threshold level.

Given defensible arguments on both sides, it was emphasized that a change in current criteria must be accompanied by assessment of the risk-benefits associated with maintaining the current criteria as compared to the public health risk-benefits if performance standards are raised.

The MPAC reviewed the need for the rigorous requirements of inter-test and inter-lot consistency, the statistical basis of repeated tests for a given number of samples from wild type parasites, the relation between PDS threshold and RDT sensitivity in well-conducted field studies, and the relevance of other important parameters such as stability at high temperature. However, they concluded that it was important that testing of RDTs against an independent panel of parasite-derived antigens be robust and mandatory, and that setting a diagnostic performance threshold measure was useful for national malaria control programmes (NMCP) as well as manufacturers, in order to encourage the development and deployment of better performing tests.

Also noted was that the fact that the majority of RDTs currently in use meet the proposed threshold of 75% PDS, so there is little likelihood that this will generate an increase in costs of the diagnostic tests. However, there is potential for increased costs at the country level to cover such activities as health care worker training and communication if the new recommendations result in a new RDT being procured.

Following discussion, the MPAC recommended a PDS threshold of 75% for *P. falciparum* at low parasite densities (200 parasites/μL) in areas of high transmission. The MPAC recommended calling on industry and development partners to make RDTs specifically for low transmission settings, and for the detection of low parasite densities in asymptomatic carriers with good stability at high temperatures in the field, congruent with the call made by the MalERA (Malaria Eradication Research Agenda) Consultative Group on Diagnoses and Diagnostics [11].

The MPAC recommended that WHO-GMP promotes the use of the on-line interactive RDT guide [12], developed by the Foundation for Innovative New Diagnostics (FIND), which allows buyers to select RDTs based on multiple factors such as target species, diagnostic performance, and stability at high temperatures, so that the RDT chosen is the one most suitable for their intended areas of use.

### Larviciding

WHO-GMP presented a draft position statement entitled: *The role of larviciding for malaria control, with particular reference to Africa*[13] prompted by a longstanding need for updated guidance on this issue, as well as current plans in several African countries for a substantial expansion of larviciding activities. The paper was developed by WHO-GMP in mid 2011, and then shared with nearly 100 experts, of whom approximately half responded. The expert opinions gathered during this consultative phase were instrumental in improving the original draft.

The MPAC noted the low volume of high quality and generalizable data with regard to larviciding, and that most vector control experts agree there are some specific circumstances where larviciding programmes can be effective and useful for malaria control (breeding sites are few, fixed and findable), but that the likely impact of this intervention may not represent a good use of limited resources and cannot substitute for indoor residual spraying (IRS) or long-lasting insecticide-treated nets (LLINs) in most settings, especially in rural areas. They agreed that for malaria vector control in Africa, it is important that NMCP managers can distinguish between situations where larviciding is likely to be useful (e.g. in selected urban settings), and those where it is inappropriate (e.g. in the majority of rural African settings). This view was supported by the two African NMCP representatives who had been invited to participate in the MPAC meeting as per its ToR [1].

The MPAC noted the recent resurgence of interest in larviciding in certain African countries. MPAC also recognized that research gaps exist and that it may be some time before there is sufficient evidence for a comprehensive policy statement on larval source management (LSM). Therefore, MPAC agreed that there is an urgent need for an interim position statement on the use of larviciding in Africa [14]. The MPAC recommended that the statement focus on larviciding in sub-Saharan Africa in particular, and that it makes clear that larviciding is not generally recommended in rural areas. It was stressed that the statement needed to balance a range of views in an area where evidence is limited. The MPAC recommended that the draft statement should be revised based on the above points with the help of a few MPAC members, before being formally issued together with a brief preamble on the rationale for an interim statement and its restriction to Africa. They also recommended that the details of the interim position statement be presented and discussed at the next ALMA meeting.

The MPAC called for a more substantive review of malaria vector control at their next meeting in September, including discussing the potential role of a malaria vector control TEG that could reflect the field's diversity and review evidence on LSM and other interventions to facilitate optimal choices of interventions within an integrated approach to malaria vector control and insecticide resistance management.

### Classification of countries for elimination

WHO-GMP presented an overview of the criteria it uses for the classification of countries by elimination phase, and the progress of countries since 2007 [15]. WHO classifies countries by the type of malaria programme that is implemented in the worst affected malaria-endemic part of its national territory. The classification, which has been published in the *World Malaria Report* since 2008, distinguishes the three distinct programme phases of control, elimination and prevention of reintroduction, and the transition phase of pre-elimination. Control-phase countries that are implementing projects aimed at achieving localized “malaria-free zones” (e.g. Hainan in China or Khartoum in Sudan) have also been listed in past editions of the *World Malaria Report*. The primary questions to the MPAC were: (a) should WHO-GMP continue to categorize countries by the type of malaria programme that is implemented in the worst affected malaria-endemic part of the country, and (b) are the current qualitative classification criteria adequate.

The MPAC was broadly in favour of continuing with a simplified classification that is useful for countries and the global malaria community to monitor progress towards elimination goals. Regional and country representatives appreciated the value of the WHO classification to help mobilize national resources and maintain momentum while also considering the economic implications regarding tourism and, in some cases, Global Fund funding. The MPAC pointed out that the country-level classification does not sufficiently capture the diversity of malaria control and elimination efforts within countries [4].

The MPAC recommended that WHO-GMP should develop an adaptation of the current classification by including programmatic as well as epidemiological determinants of country progress towards elimination for consideration at its next meeting. In addition, WHO-GMP should consider adding a country malaria risk description like the ones currently published in WHO’s *International Travel and Health* country pages [16] to the individual country profiles in the *World Malaria Report*. It was agreed that WHO-GMP will develop a proposal for a revised classification, together with draft standard operating procedures (SOP) on the certification of achievement of elimination, for presentation to the MPAC at its next meeting in September 2012.

### Estimating malaria cases and deaths

WHO-GMP presented its current methods for estimating the number of malaria cases and deaths, and compared these methods with those being used by other groups also involved in malaria burden estimation [17]. The wide uncertainty in all estimation methods, which is exacerbated by the often unknown and variable quality of the input data, in particular the lack of specificity of verbal autopsies, was highlighted. WHO-GMP works with a range of partners in the development of its estimates; however, there is still no global consensus on the best methods for malaria burden estimation given current data limitations. While it is desirable to achieve such a consensus, there also needs to be a major focus on improving diagnostic testing, surveillance, and vital registration such that the burden of malaria can be more directly measured and the information used to manage programmes. Because of these issues, WHO–GMP proposed to MPAC the establishment of an ERG to examine approaches to burden estimation with a view to identifying procedures that: (a) provide robust burden estimates around which there is consensus; (b) are open and transparent; (c) can be readily updated, e.g. changes in programme coverage; and (d) can be applied by endemic countries.

The MPAC strongly endorsed the creation of such an ERG to provide an initial report back to them by the next meeting in September 2012. Given the complex methodological discussion needed, MPAC recommended that the ToR for the ERG will need to: (a) ensure that a diversity of voices are heard; (b) focus less on past discrepancies and more on a way forward to standardize and validate methods that allow for consistent reporting of trends; (c) address how to improve the quality of the input data through improved malaria surveillance; and (d) focus on the best interests of WHO member states and the global malaria community as a whole. WHO-GMP, in close consultation with the MPAC, will quickly draft the ToR and convene an ERG with clear independence and a reporting line to the MPAC in order to make consensus-based and evidence-informed recommendations. Membership will need to balance malariologists and non-malariologists so that there is a sufficient depth and breadth of expertise.

### Seasonal malaria chemoprevention (SMC)

The co-chair of the TEG on malaria chemotherapy presented its recommendation on SMC using amodiaquine-sulphadoxine-pyrimethamine (AQ-SP) [18-20]. There is strong evidence for high efficacy (approximately 80% reduction in malaria cases) and cost-effectiveness in areas of the Sahel sub-region with marked seasonality in malaria transmission (defined as 60% of cases occurring within four months).

Subsequent discussion by the MPAC addressed questions about the choice of anti-malarial drug and appropriate pharmacovigilance measures, the practicalities of implementation in countries with artemisinin-based combination therapy (ACT) containing either AQ or SP as first-line treatment, and the potential age displacement of morbidity as a consequence of the delay in the acquisition of immunity due to the intervention. Consensus was reached on: (a) the completeness of the literature review – the general conclusion was that using the word “chemoprophylaxis”, which is a similar term to “chemoprevention”, would not have affected the outcome of the recommendation, but that it needs to be clearer why earlier studies of seasonal chemoprophylaxis were not included in the review; and (b) the effectiveness of SMC with AQ-SP – the general conclusion was that there is a window of opportunity related to the current effectiveness of AQ-SP and that SMC should be adopted soon, while operational experience and new evidence will be regularly reviewed by the MPAC.

The MPAC recommended the adoption of SMC as a new malaria control strategy pending minor changes to the policy recommendation. There was strong consensus on the need to rapidly finalize and disseminate the SMC policy recommendation, ideally within two months of the MPAC meeting.

Specific clarifications that will be made by the TEG and WHO-GMP to the SMC recommendation before MPAC endorsement include: (a) making the recommendation flexible rather than prescriptive, such that countries have latitude in how to implement this new intervention, and are not required to change their first-line treatment; (b) that methods for monitoring of effectiveness should be developed immediately; and (c) clear language regarding intervals of repeated dosing and the nature of areas and settings suitable for implementation [21].

The MPAC recommended that the implementation guide and relevant operational materials about SMC explicitly address the following issues: (a) the apparent paradox between the push for universal access to diagnostic testing for suspected malaria and the new policy on SMC; (b) the difference between SMC and other intermittent interventions e.g. Intermittent Preventive Treatment in infants (IPTi), explaining that SMC and IPTi should not be deployed simultaneously in a given area; (c) that SMC is not a replacement for existing malaria control strategies, including vector control and access to prompt diagnostic testing and effective treatment; (d) an explanation of the criteria for the literature review that provided the evidence base for SMC; and (e) an explanation of the potential age displacement of clinical malaria that may result from the intervention.

The MPAC and WHO-GMP called on product development partnerships to develop AQ-SP co-blistered combinations meeting international quality standards for use in SMC. In addition, new SMC studies should be promoted and initiated to evaluate the safety and effectiveness of combination therapies -- different from the ACT currently used for malaria treatment -- that might be used in the future for SMC in areas where AQ-SP is no longer sufficiently effective.

## Discussion

The MPAC discussed several potential topics for future meetings, mainly: (a) the management of malarial and non-malarial fevers; (b) prevention of malaria during pregnancy, including dose frequency of intermittent preventive treatment (IPTp) with sulphadoxine-pyrimethamine and if/when to stop IPTp in areas of low transmission; (c) a global strategy for the control and elimination of *P. vivax* malaria; (d) an update on the RTS,S malaria vaccine; and (e) an update on the Affordable Medicines Facility for malaria (AMFm), including the outcome of the independent evaluation of this programme. The MPAC also recommended the creation of an ERG on the use and safety of primaquine as a gametocytocide for *P. falciparum* malaria, which will report back to the MPAC at its next meeting in September, as this is an urgent issue.

The MPAC strongly encouraged engagement with and attendance by interested stakeholders at MPAC meetings. In addition to open registration for MPAC meetings, which will continue, and attendance by four standing observers (Roll Back Malaria (RBM), the Global Fund, UNICEF, Office of the UN Special Envoy for malaria) and representatives of three rotating NMCPs, WHO-GMP will continue to actively contact relevant stakeholders in the global malaria community and invite them to be observers for the next MPAC meeting in September 2012. In addition, all six WHO Regional Malaria Advisors will be invited to attend MPAC meetings as members of the Secretariat.

Standing agenda items suggested by MPAC include a brief review of ERGs and TEGs, and a review of any conditional policy recommendations or interim position statements, in case these need to be updated. In addition, MPAC meetings, to take place every March and September, fit well with RBM Board meetings, which take place every May and November. This will provide an additional mechanism for recommendations from MPAC to be disseminated to RBM partners and working groups, as well to gather feedback from RBM partners and working groups on priority issues and potential agenda items for consideration by MPAC. Feedback on agenda items will also be sought from WHO Regional Offices and NMCPs by WHO-GMP.

The MPAC suggested making malaria policies and guidelines more accessible and audience-targeted on the WHO-GMP website, an improvement that is already under consideration as part of a broader Knowledge Management Strategy by WHO-GMP, which will be shared with MPAC and other partners for input.

## Conclusions

The inaugural MPAC meeting was well attended [22] and feedback from participants and observers was very positive. The meeting marked a transition period for WHO-GMP and the global malaria community, from having no current overarching advisory body for global malaria policy setting, to a committee of experts that is engaged and committed to strengthening the policy process for malaria control and elimination. The MPAC is still in the process of orienting itself to best serve the needs of the global malaria community in responding to a rapidly evolving landscape. As such, the format of MPAC meetings and its feedback loops with other advisory bodies and stakeholders is still taking shape, and will evolve with time; WHO-GMP and the MPAC strongly welcomed feedback, support, and suggestions for improvement to MPAC meetings from the global malaria community.

Position statements and policy recommendations made by the MPAC are approved by the WHO Director General, and will be formally issued and disseminated to WHO member states by WHO-GMP. Conclusions and recommendations from MPAC meetings (following the format of this article) will be published in the *Malaria Journal* as part of this series.

The next meeting of the MPAC will take place from 11 to 13 September 2012 in Geneva, Switzerland. Further information including the agenda and details on how to register will be made available in July 2012 on the WHO-GMP website for MPAC [1].

## Endnotes

^a^The complete set of all MPAC meeting-related documents including background papers and member declarations of interest can be found online at http://www.who.int/malaria/mpac/mpacmeetings/en/.

^b^The WHO Malaria RDT Product Testing Programme is a joint project of the WHO Special Programme for Research and Training in Tropical Diseases (TDR), Foundation for Innovative New Diagnostics (FIND), US Centers for Disease Control and Prevention (CDC) and WHO-GMP, in collaboration with a number of research institutions and control programmes in malaria endemic and non-endemic countries.

^c^In product testing parasitized blood samples from patients are diluted to ensure they consistently have the same parasite density (and range of antigen concentrations); however in the field, samples of parasitized blood from patients are much more likely to have heterogeneous parasite densities -- generally with a parasitaemia higher than 200 parasites/μL.

## Abbreviations

MPAC = Malaria Policy Advisory Committee; RDT = Rapid diagnostic test; SMC = Seasonal malaria chemoprevention; WHO-GMP = World Health Organization Global Malaria Programme; ALMA = African Leaders Malaria Alliance; GPARC = Global plan for artemisinin resistance containment; TEG = Technical expert group; ERG = Evidence review group; ToR = Terms of reference; Global Fund = Global fund for AIDS, tuberculosis and malaria; PDS = Panel detection score; NMCP = National malaria control programme; malERA = Malaria eradication research agenda; FIND = Foundation for Innovative New Diagnostics; IRS = Indoor residual spraying; LLIN = Long-lasting insecticide-treated nets; LSM = Larval source management; SOP = Standard operating procedure; AQ-SP = Amodiaquine-sulphadoxine-pyrimethamine; ACT = Artemisinin-based combination therapy; IPTi = Intermittent preventive treatment in infants; IPTp = Intermittent preventive treatment in pregnancy; AMFm = Affordable medicines facility for malaria; RBM = Roll Back Malaria.

## Competing interests

The authors declare that they have no competing interests.

## Authors’ contribution

All authors listed below have equally contributed to the article. All authors have read and approved the final version of the manuscript.

## Authors’ information

WHO Malaria Policy Advisory Committee Members

· Salim Abdulla, Ifakara Health Institute, Dar Es Salaam, United Republic of Tanzania

· Pedro Alonso, Centre for International Health and Research, Barcelona, Spain

· Fred Binka, University of Ghana, Accra, Ghana

· Patricia Graves, James Cook University, Cairns, Australia

· Brian Greenwood, London School of Hygiene and Tropical Medicine, London, UK

· Rose Leke, University of Yaoundé, Yaoundé, Cameroon

· Elfatih Malik, Ministry of Health, Gezira, Sudan

· Kevin Marsh, Kenya Medical Research Institute, Kilifi, Kenya

· Sylvia Meek, Malaria Consortium, London, UK

· Kamini Mendis, Columbo, Sri Lanka

· Allan Schapira, Legazpi City, Philippines

· Larry Slutsker, Centres for Disease Control and Prevention, Atlanta, USA

· Marcel Tanner, Swiss Tropical Public Health Institute, Basel, Switzerland

· Neena Valecha, National Institute of Malaria Research, New Delhi, India

· Nicholas White, Mahidol University, Bangkok, Thailand

WHO Malaria Policy Advisory Committee Secretariat (Inaugural Meeting)

· Andrea Bosman, WHO Global Malaria Programme, Geneva, Switzerland

· Richard Cibulskis, WHO Global Malaria Programme, Geneva, Switzerland

· Valérie d'Acremont, WHO Global Malaria Programme, Geneva, Switzerland

· Jane Cunningham, WHO Special Programme for Research and Training in Tropical Diseases (TDR), Geneva, Switzerland

· Bianca D'Souza, WHO Global Malaria Programme, Geneva, Switzerland and London School of Hygiene and Tropical Medicine, London, UK

· Jo Lines, WHO Global Malaria Programme, Geneva, Switzerland and London School of Hygiene and Tropical Medicine, London, UK

· Abraham Mnzava, WHO Global Malaria Programme, Geneva, Switzerland

· Robert Newman, WHO Global Malaria Programme, Geneva, Switzerland

· Aafje Rietveld, WHO Global Malaria Programme, Geneva, Switzerland

· Peter Olemese, WHO Global Malaria Programme, Geneva, Switzerland

· Aafje Rietveld, WHO Global Malaria Programme, Geneva, Switzerland

· Pascal Ringwald, WHO Global Malaria Programme, Geneva, Switzerland
